# Neuropsychological Impairment and Its Association with Violence Risk in Japanese Forensic Psychiatric Patients: A Case-Control Study

**DOI:** 10.1371/journal.pone.0148354

**Published:** 2016-01-29

**Authors:** Hirofumi Nishinaka, Jun Nakane, Takako Nagata, Atsushi Imai, Noriomi Kuroki, Noriko Sakikawa, Mayu Omori, Osamu Kuroda, Naotsugu Hirabayashi, Yoshito Igarashi, Kenji Hashimoto

**Affiliations:** 1 Division of Clinical Neuroscience, Center for Forensic Mental Health, Chiba University, Chiba, Japan; 2 National Hospital Organization Shimofusa Psychiatric Medical Center, Chiba, Japan; 3 Department of Psychiatry, National Center of Neurology and Psychiatry, Tokyo, Japan; 4 Department of Psychiatry, Tokyo Metropolitan Matsuzawa Hospital, Tokyo, Japan; 5 Division of Law and Psychiatry, Center for Forensic Mental Health, Chiba University, Chiba, Japan; Xi'an Jiaotong University School of Medicine, CHINA

## Abstract

**Background:**

In Japan, the legislation directing treatment of offenders with psychiatric disorders was enacted in 2005. Neuropsychological impairment is highly related to functional outcomes in patients with psychiatric disorders, and several studies have suggested an association between neuropsychological impairment and violent behaviors. However, there have been no studies of neuropsychological impairment in forensic patients covered by the Japanese legislation. This study is designed to examine the neuropsychological characteristics of forensic patients in comparison to healthy controls and to assess the relationship between neuropsychological impairment and violence risk.

**Methods:**

Seventy-one forensic patients with psychiatric disorders and 54 healthy controls (matched by age, gender, and education) were enrolled. The CogState Battery (CSB) consisting of eight cognitive domains, the Iowa Gambling Task (IGT) to test emotion-based decision making, and psychological measures of violence risk including psychopathy were used.

**Results:**

Forensic patients exhibited poorer performances on all CSB subtests and the IGT than controls. For each group, partial correlational analyses indicated that poor IGT performance was related to psychopathy, especially antisocial behavior. In forensic patients, the CSB composite score was associated with risk factors for future violent behavior, including stress and noncompliance with remediation attempts.

**Conclusion:**

Forensic patients with psychiatric disorders exhibit a wide range of neuropsychological impairments, and these findings suggest that neuropsychological impairment may increase the risk of violent behavior. Therefore, the treatment of neuropsychological impairment in forensic patients with psychiatric disorders is necessary to improve functional outcomes as well as to prevent violence.

## Introduction

In Japan, the Act on Medical Care and Treatment for Persons Who Have Caused Serious Cases Under the Condition of Insanity (Medical Treatment and Supervision Act, or MTS Act) came into force on July 15, 2005, with the Ministry of Justice and the Ministry of Health, Labour and Welfare responsible for its implementation. The MTS Act encompasses individuals who have committed a serious violent offence (e.g., homicide, injury, arson, robbery, or sexual assault) while in a state of insanity or diminished responsibility. If a court panel decides to order hospitalization, the offender is detained in a designated psychiatric facility. The aim of the forensic mental health services directed by the MTS Act is to improve offenders’ reintegration in society and prevent recidivism [1].

Neuropsychological impairment, including cognitive impairment, is common in patients suffering from a variety of psychiatric disorders, and the impairment can affect multiple cognitive domains in comparison to healthy control subjects [2]. Treatment of cognitive impairment in patients with psychiatric disorders is one of the most important aspects in the field of mental health [3–7]. Neuropsychological impairment is also highly related to functional outcomes, such as life satisfaction [8], social problem solving, successful performance of daily activities [9, 10], and returning to work and school [11]. The recovery rate is inversely correlated with the severity of impairment, and even in those patients who appear to have substantially recovered, residual neuropsychological impairment compromises real-world functioning [2]. Furthermore, numerous studies using incarcerated offenders or people with antisocial or psychopathic symptoms have demonstrated that neuropsychological impairment is associated with violent behavior and that impairment in executive functioning and/or social recognition can lead to cognitive biases that increase the chances of violent behavior [12–14].

Nonetheless, there have been no studies indicating the neuropsychological characteristics in forensic psychiatric patients since the MTS Act took effect in Japan. The aims of the present study are (1) to examine the neuropsychological characteristics of Japanese forensic psychiatric patients in comparison with nonviolent healthy controls and (2) to assess the relationship between performance of neuropsychological tests and risk factors for violence, including psychopathic personality traits. This study is designed to capture broad domains of neuropsychological functioning and to assess both cognitive and emotional functions.

The Japanese-language version of the CogState Battery (CSB) and the Iowa Gambling Task (IGT) are used as measures. The CSB provides a brief standardized assessment of broad cognitive domains including verbal learning, processing speed, attention/vigilance, working memory, visual learning, reasoning and problem solving, and social cognition [15]. The IGT assesses the emotional aspects of decision making in ambiguous situations [16] and simulates real-life decision making under conditions of reward and punishment and of uncertainty [17].

## Materials and Methods

### Participants

The patients were recruited from three designated forensic hospital units that provide services under the MTS Act: the National Hospital Organization Shimofusa Psychiatric Medical Center (located in Chiba, Japan), National Center of Neurology and Psychiatry Hospital, and Tokyo Metropolitan Matsuzawa Hospital (both located in Tokyo, Japan). The healthy controls had no history of serious violence or psychiatric disorders and were recruited through an advertisement and by a dispatch service company (Souken Inc., http://www.souken-lab.co.jp/). We contacted a total of 144 individuals, of whom 125 gave informed consent to the study and complied with all procedures. The final sample comprised 71 forensic patients and 54 healthy controls.

The 71 forensic patients were diagnosed using the International Statistical Classification of Diseases, tenth revision (ICD-10; World Health Organization, 1992) by their consulting psychiatrist; the diagnoses were confirmed by another psychiatrist at a unit meeting. Nine patients were diagnosed with psychotic disorders due to psychoactive substance use (coded as F1); 61 with schizophrenia, schizoaffective disorder, or delusional disorders (coded as F2); and 1 with mood disorder (coded as F3). Twenty-three of the 71 patients had committed homicide, while 33 had been charged with injury, 11 with arson, 1 with robbery, and 3 with sexual assault. All patients were in the convalescent or rehabilitative (not the acute) stage of treatment. Four were treated with a single first-generation antipsychotic medication, 27 with a single second-generation antipsychotic medication, and 40 with a combination of antipsychotic drugs.

The 54 healthy controls were screened with clinical interviews to ensure that they did not suffer from psychiatric disorders. We made a concerted effort to recruit community participants who would match the forensic patients with regard to age, male-female ratio, smoker-nonsmoker ratio, and level of educational achievement.

Inclusion criteria for all participants in both groups included proficiency in the Japanese language, normal or corrected-to-normal visual function, and a minimum of ninth-grade education. Exclusion criteria for all participants in both the groups included any current or past histories of head injury, cerebral vascular disorders, or epilepsy.

Prior to the commencement of the study, all participants provided written informed consent after receiving a full explanation regarding the nature of the study and the potential risks and benefits of study participation. A researcher assessed their capacity to consent by three questions based on Palmer et al. [18]: (1) “What is the purpose of the study?” (2) “What are the risks?” and (3) “What are the benefits?” The individuals who had compromised capacity to consent were excluded from the study.

The study was approved by the relevant ethics committee of each institute and was performed in accordance with the Declaration of Helsinki II. The ethics committees of each institute were the Ethics Committee of Chiba University Graduate School of Medicine, the Ethics Committee of National Hospital Organization Shimofusa Psychiatric Medical Center, the Ethics Committee of National Center of Neurology and Psychiatry, and the Ethics Committee of Tokyo Metropolitan Matsuzawa Hospital.

### Demographic information

For both groups, information on sex, age, years of education, and smoking status were collected. For the forensic patients, information on duration of illness, duration of untreated psychosis, and dosage of medications was also obtained. To assess premorbid intellectual quotient (IQ), the Japanese Adult Reading Scale, which is the Japanese version of the National Adult Reading Test (JART) [19], was used with both the groups.

### Clinical measures

The World Health Organization (WHO) Quality of Life instrument (WHOQOL-26) is a 26-item, self-administered questionnaire and a shortened version of the WHOQOL-100 scale, which measures the four domains of physical health and well-being, psychological health and well-being, social relationships, and environment. Higher scores represent a better quality of life. The Positive and Negative Syndrome Scale (PANSS) was used to measure the severity of symptoms in the patients. The PANSS is a 30-item, clinician-rated instrument of positive, negative, and general psychopathology symptoms; each item is scored from 1 (absent) to 7 (severe), with a total score ranging from 30 to 210 [20].

### Violence risk measures

The Psychopathy Checklist-Revised (PCL-R) assesses inferred personality traits and behaviors related to psychopathy, using information from a semi-structured interview and records. The PCL-R consists of 20 items; each item is scored as 0 (absent), 1 (present to some degree), or 2 (fully present), with a total score ranging from 0 to 40. Factor 1 of the PCL-R measures emotional detachment, lack of empathy and remorse, fearlessness, and insensitivity to punishment, whereas Factor 2 covers impulsiveness and antisocial lifestyle [21].

For the forensic patients, the Historical Clinical Risk Management-20 (HCR-20) was also used to assess violence risk based on information from a semi-structured interview and records. The HCR-20 includes 20 items and 3 subscales; each item is scored as 0 (not present), 1 (possibly or partially present), or 2 (definitely present), with a total score ranging from 0 to 40. Ten items relate to historical (H) or static risk factors (e.g., previous violence, age at first violent incident), five cover clinical (C) or current risk factors (e.g., lack of insight, impulsivity), and five concern risk management (R) or future-oriented factors (e.g., lack of personal support, stress) [22].

The CogState Battery (CSB), Japanese-language version, is a rapid, automatically administered, computerized battery that assesses verbal learning and memory (using the International Shopping List Task, or ISLT), visual learning and memory (One Card Learning Task, OCL), speed of processing (Detection Task, DET), attention and vigilance (Identification Task, IDN), visual working memory (Two Back Task, TWOB), spatial working memory (Continuous Paired Association Learning Task, CPAL), reasoning, problem solving, and error monitoring (Groton Maze Learning Task, GML), and social cognition (Social Emotional Cognition Task, SECT) [15]. These tasks were presented on a green screen, along with standardized instructions given by a trained researcher before the commencement of each task, to ensure that all participants completely understood and followed the rules. The results were uploaded to a secure account on the CogState server site (http://www.cogstate.com), where data were calculated and normalized. The primary measure from each task of the CSB was standardized by creating Z-scores. The mean for the control group was set at zero and the standard deviation at one, following the methodological procedure used by Keefe et al. [23]. A composite score was calculated by averaging all Z-scores from the eight primary measures contained in the CSB.

The IGT was described in detail in a previous study [16]. Briefly, the task goal is to maximize the profit from a loan granted in play money. The participant is required to make a series of 100 card selections from one of four card decks (A, B, C, and D). Each selection is followed by the showdown of a reward and a penalty. The reward and penalty schedules are predetermined but not explained to the participant in advance. Decks A and B yield high immediate rewards but carry the risk of much higher long-term penalties, which will result in a net loss in the long run; they are thus referred to as disadvantageous decks. Decks C and D yield small immediate rewards but even smaller long-term penalties, resulting in a net long-term gain (and making them advantageous decks). We developed a computerized Japanese version of the IGT in strict compliance with the original version [24]. The only substantive difference from the original task was that the play money was converted from U.S. dollars to Japanese yen. After they completed the task, the participants were asked which decks they considered advantageous. IGT performance was described as a net score calculated by subtracting the number of cards selected from the two disadvantageous decks (A + B) from the number selected from the two advantageous decks (C + D). Higher scores reflected more advantageous decision-making performance on the task.

### Statistical analyses

SPSS for Windows, version 19 (SPSS Inc., Chicago, USA), was used for all analyses. Student’s *t*-test and Fisher’s exact test were used to examine differences between groups. For comparison of the IGT scores between groups, a two-way repeated ANOVA (2 groups × 5 blocks of 20 trials) was performed, and multiple analyses by post-hoc Bonferroni testing were used. ANCOVAs were performed if potential relationships between demographic data and scores of neuropsychological, clinical, or violence risk measures were observed in preliminary correlational analyses. Partial correlational analyses were performed separately for the forensic and control groups, respectively, to evaluate relationships between neuropsychological test performance and violence risk scores. Demographic and clinical variables were controlled because of the possibility that these variables might affect neuropsychological functions and violence risk. For the healthy group, the controlled variables included age, sex, years of education, smoking status, premorbid IQ, and QOL score; for the patient group, all these variables plus duration of illness, duration of untreated psychosis, dosage of medications, and PANSS total score were controlled. Values of *p* < 0.05 were considered as indicating statistical significance.

## Results

### Comparing forensic patients with controls on demographic, neuropsychological, clinical, and violence risk measures

Information on demographic and clinical domains and violence risk in both forensic patients and healthy controls is presented in Table 1. On the demographic measures, a series of *t*-tests and Fisher’s exact tests indicated that the two groups were matched for age (*p* = 0.729), sex (*p* = 0.601), years of education (*p* = 0.329), and smoking status (*p* = 1.000), but the mean premorbid IQ in the forensic patients was significantly lower than in the controls (*p* < 0.001). To estimate the potential relationship of premorbid IQ to violence risk and clinical and neuropsychological measures, the correlations were calculated on the whole sample (N = 125) before comparing the two groups on these measures. There were significant correlations between premorbid IQ and scores on all measures (QOL, *r* = 0.37, *p* < 0.001; PCL-R, *r* = −0.29, p = 0.001; CSB composite score, *r* = 0.49, *p* < 0.001) except IGT net score (*r* = 0.15, *p* = 0.95).

**Table 1:** Characteristics of demographic and clinical domains and violence risk in forensic patients and healthy controls.

	Controls (n = 54)	Patients (n = 71)	Statistics	*p* value
**Demographic domains**				
Age (years)	42.06 ± 11.43 (23–69)	42.79 ± 11.92 (21–74)	*t* = -0.35^a^	0.729
Sex (male/female)	48/6	60/11	*χ*^*2*^ = 0.50^b^	0.601
Education (years)	12.76 ± 2.66 (9–18)	12.30 ± 2.58 (9–21)	*t* = 0.98^a^	0.329
Smoking status (current/non-smoker)	32/22	41/30	*χ*^*2*^ = 0.03^b^	1.000
Premorbid IQ	106.04 ± 9.83 (86–122)	99.45 ± 10.69 (78–120)	*t* = 3.53^a^	< 0.001
Duration of illness (years)		18.07 ± 9.87 (4–43)		
Duration of untreated psychosis (years)		4.08 ± 5.92 (0–25)		
**Dosage of medications**				
Chlorpromazine equivalents (mg)		756.97 ± 598.22 (13–2902)		
Diazepam equivalents (mg)		11.33 ± 13.80 (0–70)		
Biperiden equivalents (mg)		1.70 ± 2.33 (0–12)		
**Clinical domains**				
WHO-QOL26 score	3.36 ± 0.51 (1.62–4.23)	2.98 ± 0.57 (1.69–4.88)	*F* = 7.88^c^	0.006
PANSS total score		56.97 ± 19.59 (30–117)		
**Violence risk**				
PCL-R total score	5.24 ± 3.96 (0–18)	11.25 ± 4.72 (1–23)	*F* = 45.39^c^	< 0.001
HCR-20 total score		18.82 ± 4.12 (10–27)		

Data are the mean ± S.D. Parenthesis is the range.

^a^ Student's t-teat

^b^ Fisher's exact test

^c^ ANCOVA with premorbid IQ as a covariable

Due to the potential effect of premorbid IQ on QOL and PCL-R scores, ANCOVAs with premorbid IQ as a covariable were conducted. In this analysis, the forensic patients had more severe problems on the QOL (*F* = 7.88, *p* = 0.006) and higher scores on the PCL-R (*F* = 45.39, *p* < 0.001) than the controls (Table 1).

**Fig 1:**
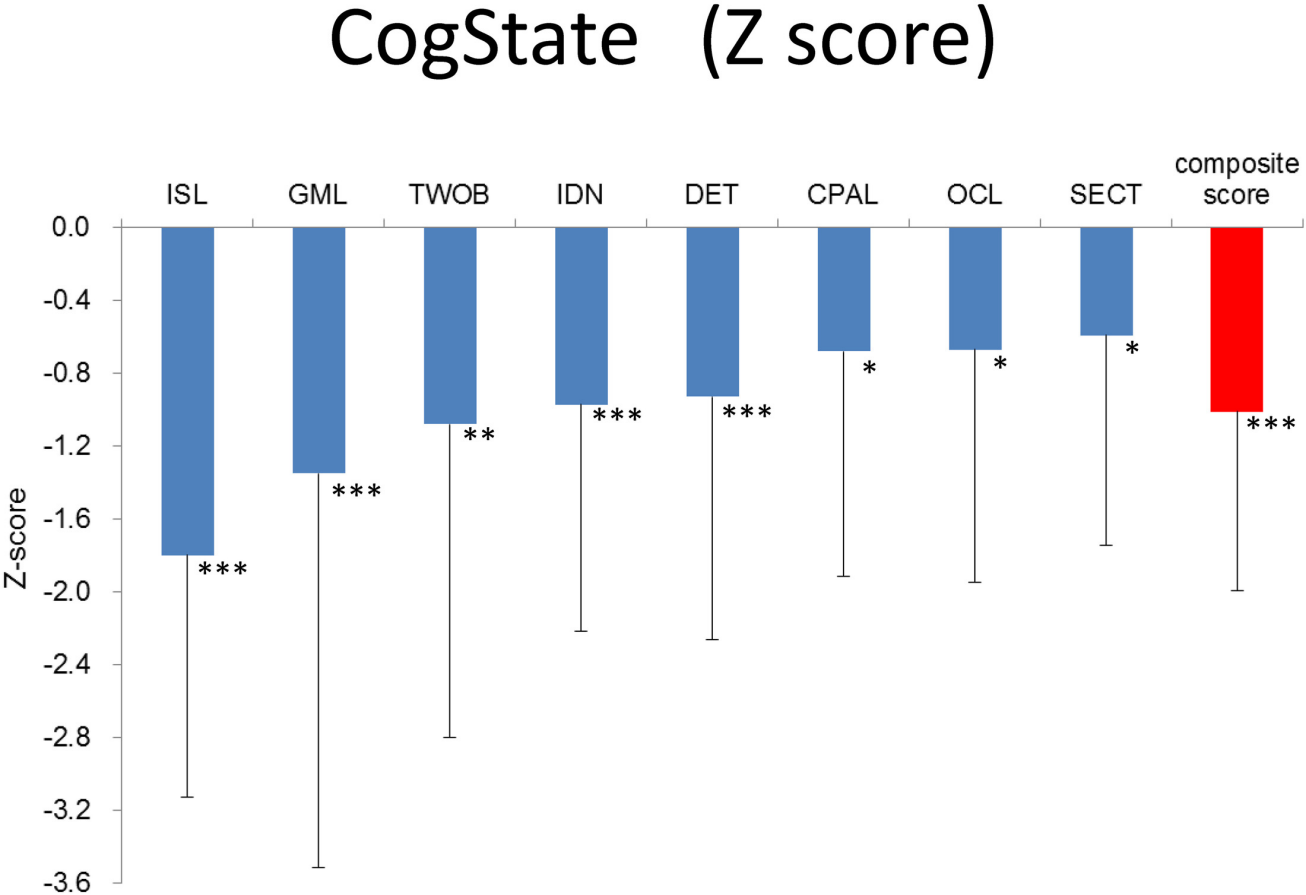
Magnitude of impairment in forensic patients relative to healthy controls on each CSB measure. Mean ± SD of Z-scores are given. Z-score was created by setting controls’ mean to zero and SD to one. Abbreviation: ISL International Shopping List Task, GML Groton Maze Learning Task, TWOB Two Back Task, IDN Identification Task, DET Detection Task, CPAL Continuous Paired Association Task, OCL One Card Learning Task, SECT Social Emotional Cognitive Task. * *p* < 0.05, ** *p* < 0.01, *** *p* < 0.001.

With regard to the CSB, the ANCOVAs with premorbid IQ as a covariable indicated that the scores on the ISL (*F* = 51.86, *p* < 0.001), TWOB (*F* = 8.94, *p* = 0.003), IDN (*F* = 16.44, *p* < 0.001), DET (*F* = 11.82, *p* < 0.001), CPAL (*F* = 4.45, *p* = 0.037), OCL (*F* = 5.95, *p* = 0.016), and SECT (*F* = 5.72, *p* = 0.018), as well as the composite score (*F* = 29.85, *p* < 0.001), were lower in forensic patients than in controls. Since regression lines of group factor and premorbid IQ to the GML score were not parallel (group—premorbid IQ interaction was significant), a *t*-test for the GML score was performed without premorbid IQ as a covariable. Forensic patients had lower scores on the GML (*t* = 4.63, *p* < 0.001), indicating that they exhibited poorer performances on all CSB domains than controls (Fig 1).

**Fig 2:**
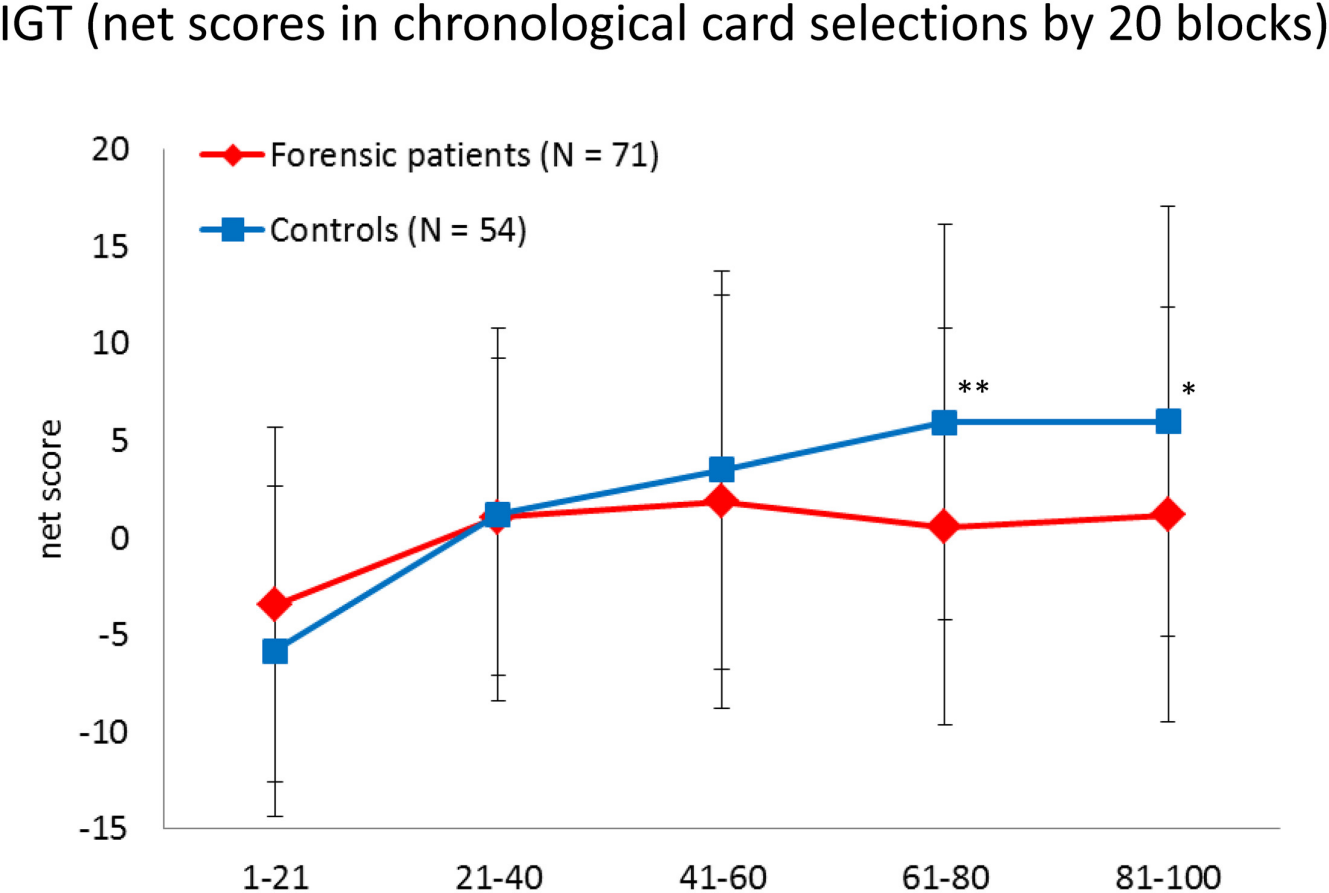
The IGT net scores for the 5 blocks for forensic patients and healthy controls. Mean ± SD are given. * *p* < 0.05, ** *p* < 0.01.

As for performance on the IGT, a two-way repeated ANOVA (2 groups × 5 blocks of 20 trials) was conducted without premorbid IQ as a covariable due to the lack of correlation between the two in the preliminary correlation analysis. The ANOVA demonstrated a significant main effect for blocks (*F* = 16.51, *p* < 0.001), with participants becoming increasingly risk-aversive over time. A primary effect for groups was marginally significant (*F* = 3.90, *p* = 0.051). The block—group interaction was significant (*F* = 3.30, *p* = 0.011), with controls indicating a greater tendency to become more risk-aversive over time than forensic patients. Post-hoc Bonferroni analysis showed that forensic patients differed significantly from controls in block 4 (*p* = 0.004) and in block 5 (*p* = 0.022) (Fig 2).

Furthermore, we examined the effect of diagnosis categories or criminal types on neuropsychological domains, although the number of participants in some diagnosis categories or criminal types was small (data not shown). Due to the very small number of participants, patients coded as F3 (n = 1) or who committed robbery (n = 1) or sexual assault (n = 3) were excluded from each analysis. F1 patients (n = 9) exhibited lower scores than the control group on the IDN, ISL, and CSB composite score, and F2 patients (n = 61) had lower scores than controls on all neuropsychological domains except the CPAL, SECT, and IGT net score. There were no differences between F1 and F2 patients on all tests. Patients who had committed homicide (n = 23) were significantly lower than controls on the DET, IDN, TWOB, ISL, and CSB composite score; the injury group (n = 33) had lower IDN, GML, ISL, CPAL, and composite scores; the arson group (n = 11) had lower IDN and ISL scores than controls. Furthermore, there were no differences among these three criminal types on all tests.

### Partial correlation analyses between neuropsychological functions and violence risk

Partial correlation analyses were performed with the data from both groups, controlling for demographic and clinical variables. For the control group, Table 2 indicates significant negative correlations between IGT performance and both PCL-R Factor 1 (*r* = −0.29, *p* = 0.047) and Factor 2 (*r* = −0.35, *p* = 0.018). With regard to the forensic patients, Table 3 shows a significant negative correlation of IGT performance with PCL-R Factor 2 (*r* = −0.30, *p* = 0.031). These results indicated that participants with high PCL-R scores exhibited more risky decision making in an ambiguous situation. Negative correlations between the CSB composite score and both the PCL-R Factor 2 score (*r* = −0.27, *p* = 0.054) and HCR-20 R score (*r* = −0.27, *p* = 0.052) were marginally significant in forensic patients.

**Table 2:** Partial correlations between scores of measures on violence risk and neuropsychological performances in healthy controls.

	CSB composite score		IGT net score	
Measures	*r*	*p* value	*r*	*p* value
**PCL-R total**	-0.07	0.660	-0.37	0.010
Personal/affective factor (Factor 1)	0.14	0.355	-0.29	0.047
Antisocial deviant factor (Factor 2)	-0.19	0.188	-0.35	0.018

Partial correlation coefficients were calculated after controlling for age, sex, education years, smoking status, premorbid IQ, and QOL score.

**Table 3:** Partial correlations between scores of measures on violence risk and neuropsychological performances in forensic patients.

	CSB composite score		IGT net score	
Measures	*r*	*p* value	*r*	*p* value
**PCL-R total**	-0.13	0.346	-0.28	0.045
personal/affective factor (Factor 1)	-0.10	0.482	-0.08	0.582
antisocial deviant factor (Factor 2)	-0.27	0.054	-0.33	0.017
**HCR-20 total**	-0.12	0.407	-0.16	0.258
Historical (H) factor	-0.03	0.807	-0.01	0.932
Clinical (C) factor	-0.01	0.973	-0.19	0.165
Risk management (R) factor	-0.27	0.052	-0.16	0.242

Partial correlation coefficients were calculated after controlling for age, sex, education years, smoking status, premorbid IQ, illness duration, duration of untreated psychosis, the dosage of medications, QOL score, and PANSS total score.

## Discussion

This is the first study to investigate neuropsychological characteristics and their associations with violence risk in forensic psychiatric patients covered by the MTS Act in Japan.

We used the PCL-R and HCR-20 for violence risk assessment. Zhou’s review suggested that the validity of these instruments developed in the West is poorer for Chinese samples than that for Western ones [25]. However, their review included only two PCL-R and three HCR-20 studies also estimated the validity only in Chinese samples but not in other Asian samples, including Japanese ones. Furthermore, the PCL-R can predict aggression in Korean inmates [26], and the HCR-20 demonstrates similar predictive accuracy across Asian-American (including Japanese), Native Hawaiian, and Euro-American samples [27]. Taken together, we believe that the PCL-R and HCR-20 could be applicable for Asian samples, including Japanese ones.

Forensic patients exhibited higher scores of violence risk (illustrated by higher PCL-R scores) although their mean PCL-R score did not exceed 30, which is considered the cut-off point for the label of psychopathy. This difference between the groups was not surprising, as approximately 20–30% of patients with schizophrenia have psychopathic traits in foreign forensic psychiatric settings [28, 29]. Accumulating evidence suggests that individuals with psychiatric disorders are at increased risk for violent offending, relative to the general population [30, 31]. The QOL score was also lower for the forensic group. Schizophrenic patients are thought to be less satisfied than other persons in various QOL domains due to the mental illness itself, psychopathological symptoms, and psychosocial factors [32–34]. Substance abuse and psychiatric comorbidity are also associated with impaired QOL [35].

Forensic psychiatric patients had broader and more severe cognitive problems as assessed by the CSB. In Japanese patients with schizophrenia, Yoshida et al. [15] reported similar results when using the CSB. The findings of meta-analyses have indicated that cognitive impairment in patients with schizophrenia is evident in general functioning and across a range of cognitive domains [2, 36, 37]. Thus, cognitive impairment is a core feature of schizophrenia. Substance abuse also negatively has impacts on cognitive functioning [38, 39].

Moreover, forensic patients exhibited poorer decision making on the IGT than the control group. This finding is supported by most of the literature on patients with schizophrenia or substance abuse [40–44]. In accordance with previous reports [45, 46], forensic patients in our samples indicated lower net scores in chronologically later blocks over the duration of the IGT than controls. Forensic patients were less likely to avoid making risky selections during the task, suggesting that they may fail to learn from emotional feedback. Deficits in clinical and neuropsychological domains among these forensic psychiatric patients are consistent with deficits found in general psychiatric patients.

Although the present study did not compare forensic with non-forensic psychiatric patients, several studies have made this comparison [14, 30]. The results of these previous studies are inconsistent; some studies showed that forensic patients with mental disorders (mostly persons with schizophrenia) had more severe impairment of executive functioning [47–49] and general cognitive functioning [50] than non-forensic counterparts. Silver et al. [51] found that forensic patients with schizophrenia showed poorer ability to discriminate between intensity levels of facial emotion than their non-violent counterparts. From the present study, it is unknown whether their performances were different from those of non-forensic patients and whether these are related to violence or psychiatric disorders. It should be noted that other variables, including the use of medication and substances, might impact neuropsychological performance.

Next, partial correlation analyses were performed to examine the relationship between neuropsychological function and violence risk. Demographic and clinical variables were controlled because of the possibility that these variables might affect neuropsychological functions and violence risk. For the forensic patients, these variables included medication dosages, since all patients were in the convalescent or rehabilitative stage of treatment. This is the first investigation to demonstrate an association between psychopathy and decision making on the IGT in forensic psychiatric patients. Even when demographic and clinical variables were controlled, poor decision making in both groups was related to psychopathy. Individuals with psychopathic traits may be more likely to make risky decisions in ambiguous situations, and they fail to learn from emotional feedback to adjust their deviant behavior, including violence; this relationship appeared in both groups in our sample. Furthermore, poor decision making was more related to the antisocial deviance factor (i.e., PCL-R Factor 2) than to the personal/affective factor (PCL-R Factor 1). These results were similar to those in previous studies of inmates or nonclinical individuals [52–55]. The psychopathic characteristics of antisocial deviance, quick temper, and explosive anger tend to have a relatively strong association with poor decision making [53, 55]. Performance deficits observed by using the IGT have been linked to lesions in the ventromedial prefrontal cortex (vmPFC) [16]. In addition, individuals with vmPFC damage commonly display a syndrome that encompasses poor judgment, socially inappropriate behavior, and impulsivity [56]. The IGT is also associated with emotion-based decision making and separable from cognitive abilities [57]. Alternatively, an association between the CSB composite score and the PCL-R’s antisocial deviance factor was marginally significant in forensic patients. These results suggest that psychopathic antisocial behavior is more likely to be involved in emotional rather than in cognitive processes, although the possibility of an association between psychopathic behavior and cognitive processes cannot be ruled out.

With regard to the other measure of violence risk, the HCR-20, the association between the CSB composite score and HCR-20 R score was marginally significant, whereas the association with IGT performance was not significant in forensic patients. There may be a relationship between violence risk and cognitive impairment as measured by the CSB, although the evidence from the present results was insufficient. The R scale is related to future risk factors, including exposure to destabilizers, stress, and noncompliance with remediation attempts. As Weiss [14] pointed out in her review, limitations in executive functioning and/or social recognition would lead to cognitive biases that increase the chances of violence in response to stressful and provocative situations.

The present findings imply that different types of neuropsychological impairment may lead to violence risk through different but partially overlapping pathways among forensic patients with psychiatric disorders. This study suggests that deficits in emotional processes related to decision making may contribute to psychopathic antisocial behavior. Furthermore, cognitive impairment can cause inadequate responses to stressful and provocative situations. As violence can be distinguished between the reactive and instrumental domains, future studies should examine more specific associations between the two violence domains and neuropsychological functioning.

The present study has several limitations. First, it did not compare forensic patients with their non-forensic counterparts; therefore, it is unclear whether non-forensic patients would exhibit any different neuropsychological and clinical status and whether these are related to violence or psychiatric disorders. Second, the group of forensic patients was heterogeneous, consisting of 9 patients coded as F1, 61 coded as F2, and 1 coded as F3 on the ICD-10. The F1 group included individuals with psychotic disorders due to the use of alcohol, cannabinoids, volatile solvents, hallucinogens, or multiple drugs, and the F2 group included persons with schizophrenia, schizoaffective disorder, or delusional disorder. Further study using a larger sample size for each group is needed. Finally, this was a cross-sectional study, and further prospective studies will be needed.

In conclusion, the present study has found that forensic patients with psychiatric disorders have a wide range of neuropsychological impairments that result in poor functional outcomes. Furthermore, poor emotional decision making was related to psychopathy, especially antisocial behavior. Cognitive impairment in forensic patients may also be associated with violence risk. Therefore, the treatment of neuropsychological impairment in forensic patients with psychiatric disorders is necessary to improve functional outcomes and to prevent violence. Moreover, understanding the characteristics of a wide variety of types of neuropsychological impairment is critical to the development of suitable treatment strategies for each forensic patient.
